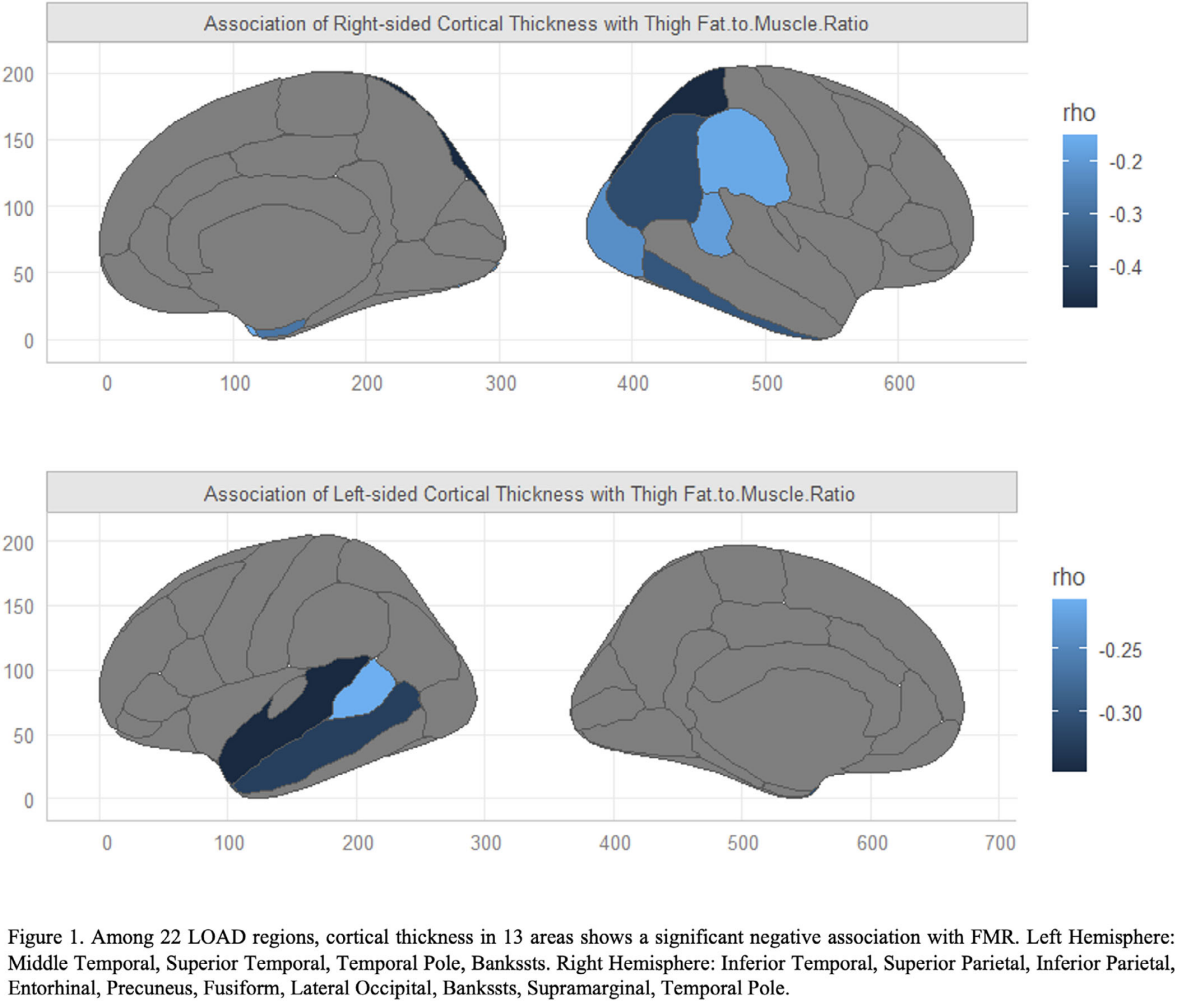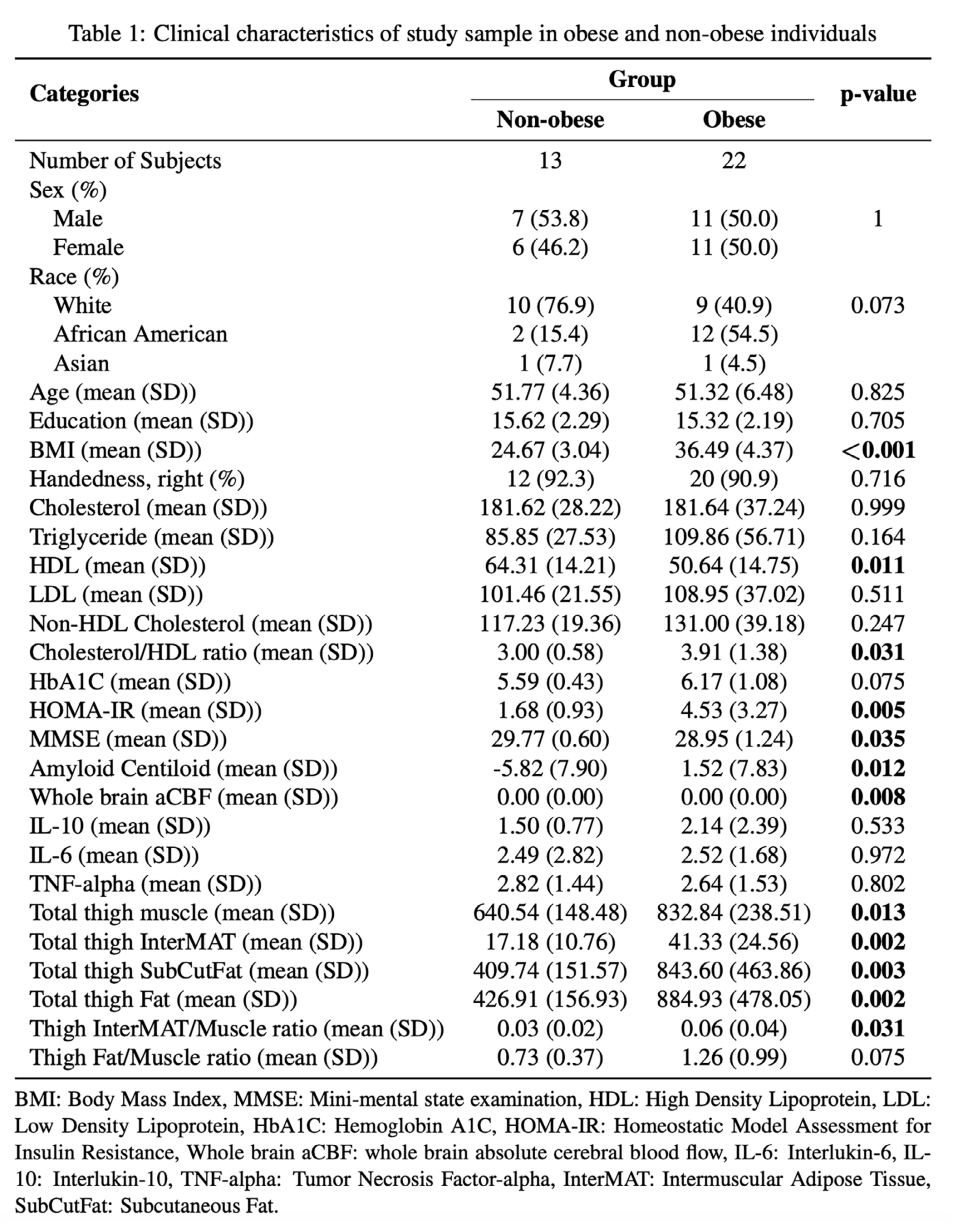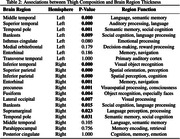# Fat to muscle ratio is related to neurodegeneration in midlife obesity

**DOI:** 10.1002/alz.092464

**Published:** 2025-01-09

**Authors:** Mahshid Naghashzadeh, Mahsa Dolatshahi, Paul K. Commean, Sara Hosseinzadeh Kasani, Farzaneh Rahmani, Jingxia Liu, LaKisha Lloyd, Caitlyn Nguyen, Nancy Hantler, Abby McBee‐Kemper, Maria Ly, Gary Z Yu, Joseph E. Ippolito, Jake Weeks, Lael Ceriani, Claude Sirlin, John C. Morris, Tammie L.S. Benzinger, Cyrus A. Raji

**Affiliations:** ^1^ Washington University in St. Louis, St. Louis, MO USA; ^2^ Mallinckrodt Institute of Radiology, Washington University in St. Louis, St. Louis, MO USA; ^3^ Washington University in St. Louis School of Medicine, St. Louis, MO USA; ^4^ University of California, San Diego, La Jolla, CA USA; ^5^ Washington University in St. Louis, School of Medicine, St. Louis, MO USA; ^6^ Knight Alzheimer Disease Research Center, St. Louis, MO USA

## Abstract

**Background:**

Emerging research underscores the significance of midlife obesity, defined by a BMI of 30 kg/m^2^ or higher in persons age 40‐60 years, as a risk factor for Alzheimer's disease (AD) in later life. Due to the various properties of each body component, it is important to characterize the neurodegenerative effects of fat within the muscle, known as a predictor of metabolic health and cognition. We investigated the relationships between thigh total fat‐to‐muscle ratio (FMR) and brain cortical thickness in cognitively normal midlife individuals.

**Method:**

Our study focused on a sample of 35 cognitively normal midlife participants (age: 51.59±5.72 years; 42.9% male; 60% obese; average BMI: 32.04±6.98 kg/m^2^). The brain and thigh scans were obtained from Siemens 3T MR scanners. On thigh MRI nine mid‐thigh slices located between Ischial Ramus and the medial knee condyle were identified and preprocessed. N4ITK Bias correction was performed with 3D Slicer to correct inhomogeneities. An in‐house MATLAB program measured total fat (subcutaneous, inter‐, and intra‐muscular fat) and muscle volumes derived from the summation of compartments from both thighs to calculate total FMR. FreeSurfer 7.1.1 was utilized for automated segmentation of both cortical and subcortical brain regions using a probabilistic atlas followed by a visual review, and where necessary, adjustments were made manually. The association between FMR and cortical thickness in 22 Late‐onset Alzheimer disease (LOAD) brain regions was examined using Spearman rank test, adjusted for age and sex and multiple comparisons.

**Result:**

FMR in the thigh is highly correlated to BMI, but not insulin resistance. There was a negative association between both FMR and InterMAT/muscle ratio and cortical thickness in multiple LOAD regions. Out of 22 LOAD regions, FMR showed negative correlation with cortical thickness in right superior and inferior parietal, inferior temporal, supramarginal, entorhinal, and precuneus cortex, as well as left middle and superior temporal lobe, and temporal pole.

**Conclusion:**

Thigh fat/muscle ratio is highly associated with BMI and negatively correlated with cortical thickness across various AD‐related brain regions. This may suggest that interventions that improve muscle quality, by reducing fat surrounding and inside the muscle, may be preventive for Alzheimer disease risk.